# Multivariate analysis differentiates intertemporal choices in both value and cognitive control network

**DOI:** 10.3389/fnins.2023.1037294

**Published:** 2023-02-28

**Authors:** Yuting Ye, Yanqing Wang

**Affiliations:** ^1^Institute of Psychology, School of Public Affairs, Xiamen University, Xiamen, China; ^2^Institute of Psychology, Chinese Academy of Sciences, Beijing, China; ^3^Department of Psychology, University of Chinese Academy of Sciences, Beijing, China

**Keywords:** intertemporal choice, multivariate analysis, value network, cognitive control network, individual differences

## Abstract

Choices between immediate smaller reward and long-term larger reward are referred to as intertemporal choice. Numerous functional magnetic resonance imaging (fMRI) studies have investigated the neural substrates of intertemporal choice via conventional univariate analytical approaches, revealing dissociable activations of decisions involving immediately available rewards and decisions involving delayed rewards in value network. With the help of multivariate analyses, which is more sensitive for evaluating information encoded in spatially distributed patterns, we showed that fMRI activity patterns represent viable signatures of intertemporal choice, as well as individual differences while controlling for age. Notably, in addition to value network, regions from cognitive control network play prominent roles in differentiating between different intertemporal choices as well as individuals with distinct discount rates. These findings provide clear evidence that substantiates the important role of value and cognitive control networks in the neural representation of one’s intertemporal decisions.

## Introduction

Choices between the temptation of immediate gratification and better long-term outcome are ubiquitous in our daily lives. The ability to forgo an immediate reward in order to achieve another goal can predict one’s cognitive, coping, and social competency (Mischel et al., 1988). Such individual trait is widely assessed by a paradigm called intertemporal choice task, which requires participants to repeatedly choose between receiving a smaller amount of money immediately or a larger amount of money later. Substantial individual differences in intertemporal choice task have been reported, which are indexed by the delay discount rate, with higher rate representing the preference for immediate rewards at the cost of larger future rewards. One’s discount rate is very stable across time (Ohmura et al., 2006; Kirby, 2009; Senecal et al., 2012), correlated to intelligence and working memory (Shamosh et al., 2008). Evidence from clinical populations has linked abnormal discount rate to various psychiatric disorders such as substance abuse (Bickel et al., 1999) and pathological gambling (Alessi and Petry, 2003).

Efforts have been made to investigate the cognitive and neural mechanistic bases of one’s intertemporal choice (Peters and Buchel, 2011; van den Bos and McClure, 2013). Several lines of empirical evidence have consistently suggested the involvement of value system, where value is computed and predicted, such as ventromedial prefrontal cortex (vmPFC), ventral striatum, and posterior cingulate cortex (PCC). Behaviorally, the value of reward is discounted as a function of the delay to receiving it, paralleled neural activities are found in vmPFC, ventral striatum, and PCC, as they preferentially respond to immediate rewards over the delayed ones (McClure et al., 2004, 2007), and damage to these areas leads to steep discounting of later rewards (Ciaramelli et al., 2021). Meanwhile, regions in cognitive control network, which support a broad range of executive functions, such as dorsolateral prefrontal cortex (dlPFC) (Niendam et al., 2012) are uniformly activated at all decision epochs independent of the delay (McClure et al., 2004; Ballard et al., 2017). Nevertheless, self-control has been suggested as a critical component of integrative framework of intertemporal choice (Berns et al., 2007). It has been shown that prefrontal control areas are recruited for difficult decisions as compared to the easy ones (McClure et al., 2004; Ballard et al., 2017; Jimura et al., 2018). Their functional couplings with reward networks in resting state successfully predicte one’s discount rate (Wang et al., 2020), and stimulation of dlPFC decreases one’s discount rate (He et al., 2016). After all, patience calls for the ability to wait in addition to the desirability of the reward. Besides valuation network and cognitive control network, recent studies also indicate a link between activities in episodic prospection network (e.g., amygdala, parahippocampus gyrus, insula) and one’s decision of intertemporal choices (Chen et al., 2019). These previous efforts suggest that instead of one or two single regions alone, brain regions from multiple networks are likely to work in concert in one’s intertemporal choice.

Most existing works investigated the neural substrate of intertemporal decision-making via conventional univariate analytical approaches. Despite being a powerful tool to investigate structure-function mappings when activations differ in local peaks or clusters of activity (Friston et al., 1994), univariate analysis is insensitive to information represented in a distributed manner across voxels (Gluth et al., 2012). Meanwhile, specific decision-making can be encoded in a spatially distributed way (Gherman and Philiastides, 2018; Yau et al., 2020). This kind of distributed information can be measured with the help of multivariate approach, which overcomes the limitations of univariate approach by searching for the optimal combination of scattered voxels and evaluating their contributions to decision discriminability (Kriegeskorte et al., 2006; Davis et al., 2014). However, albeit being widely employed in studying individual’s perceptual and cognitive status (Haxby, 2012), multivariate analysis has relatively rarely been implemented in the functional imaging of intertemporal choice (Wang et al., 2014, 2021; Chen et al., 2019; Piva et al., 2019), with most studies focusing on the dissociable representations of different intertemporal choices. Meanwhile, individual differences in intertemporal choice task have seldom been probed by this approach, especially with task-based images. In view of the stability of the discount rate and its correlation with one’s cognitive ability and mental health (Bickel et al., 1999; Alessi and Petry, 2003; Shamosh et al., 2008), it’s important to investigate the neural correlates that explain interindividual variability in discount rate.

In this study, we combined functional magnetic resonance imaging (fMRI) with multivariate analysis techniques in a relatively large sample to measure spatial ensemble coding of different intertemporal choices, as well as of the same choice contrast from individuals with different discount rates, which has been investigated to a lesser extent. Specifically, we trained classifiers to test whether and where the ensemble fMRI activity patterns represent viable signatures of intertemporal choice and individual differences, respectively. In addition to value network whose dissociable activations of different choices have been well-established by prior univariate analyses, we particularly paid attention to the recruitment of cognitive control network.

## Materials and methods

### Participants

The data used in this study were obtained from a Cognitive Training Study via the OpenNeuro database (accession number: ds002843). The data set contains behavioral and brain imaging data from 166 healthy adults. Full details regarding the original participant recruitment, exclusions, and study procedures can be found in the corresponding paper (Kable et al., 2017). In brief, all participants completed two fMRI testing sessions on two separate days, before and after cognitive training, respectively. At each session, participants completed four runs of intertemporal choice task. For the purpose of this study, the current analysis only included data from the pretraining session.

After removing participants with missing files, extreme choice behaviors (discount rate, k < 0.0017 or k > 0.077, based on a previous work (Kable et al., 2017), due to the strong imbalance between small sooner reward options and larger later reward options) and large head motions (>3 mm of translation or 3° of rotation), fMRI data from a total of 142 participants were included (57 females, mean age ± SD = 24.58 ± 4.42 years).

Moreover, to examine whether brain activity patterns represent dissociable signature of individual differences of intertemporal choice (indexed by discount rate), a subset of participants (*n* = 84) were chosen as high (*n* = 42, 18 women, 25.63 ± 4.36 years) and low impulsive group (*n* = 42, 18 women, 23.48 ± 4.42 years) based on their discount rate (detailed description in Behavioral data analysis section). In addition, to control for the influence of age, this subset of participants (*n* = 84) were regrouped in half (*n* = 42 for each group) based on their age, i.e., a median split of the participants by age (for younger and older group respectively: 19 and 17 female, mean age ± SD = 20.83 ± 2.19 and 28.26 ± 2.80).

All participants gave written informed consent following procedures approved by the University of Pennsylvania Institutional Review Board.

### Intertemporal choice task

The detailed description of the intertemporal choice task has been provided elsewhere (Kable and Glimcher, 2009). In brief, participants had to choose between a fixed small sooner reward (SS, $20 received today) and a larger later (LL, e.g., $40 in a month, range of amount: $21∼$58; rang of duration: 2∼180 days) reward, whose magnitude and delay varied from trial to trial. After a choice was made, choice feedback (1 s) was given to the participants. Participants had 4 s to make their choice. For each session, participants completed a total of 120 intertemporal choices inside the scanner.

### fMRI data acquisition

Magnetic resonance imagingscans were performed using a Siemens Trio 3 T scanner and a Siemens 32-channel head coil optimized for parallel imaging. A standard echo-planar imaging (EPI) sequence was used to acquire BOLD fMRI data while participants performed the intertemporal choice task (voxel size: 3 mm × 3 mm × 3 mm; matrix: 64 × 64; axial slices: 53 axial slices; TR: 3,000 ms; TE: 25 ms). High-resolution anatomical images were obtained using a standard Magnetization Prepared Rapid Acquisition Gradient Echo (MPRAGE) sequence (TR: 1,100 ms; axial slices: 160 axial slices; matrix: 192 × 256). Additionally, a B0 field map was acquired (TR: 1270 ms; TE1: 5.0 ms; TE2: 7.46 ms) to support the off-line estimation of geometric distortion in the functional data.

### Behavioral data analysis

Discount rates (k) in this experiment were estimated by fitting a logistic regression to data (Kable et al., 2017). Higher values of k indicate greater discounting of monetary value over time and less tolerance of delay. Since the original delay discount rate was not normally distributed, a log10 transformation was applied [log (k)]. Participants with discount rate in the top 30% of the distribution were assigned to the high impulsive group whereas those with discount rate in the bottom 30% to the low impulsive group.

### fMRI data analysis

The preprocessing of fMRI data was done using the SPM12 software^1^ on the MATLAB platform. Functional images were spatially realigned to the first image in the time series, and were corrected for movement-related variance based on the field map and movement-by-distortion interactions using the Unwarp tool in SPM. The T1 structural image was co-registered to the mean aligned functional image, and underwent segmentation and spatial normalization to MNI space. Realigned functional images were normalized using the transformation parameters derived from the structural image normalization. Finally, the normalized functional images were smoothed with a 6mm full-width half-maximum Gaussian kernel (Vermeylen et al., 2020).

**FIGURE 1:**
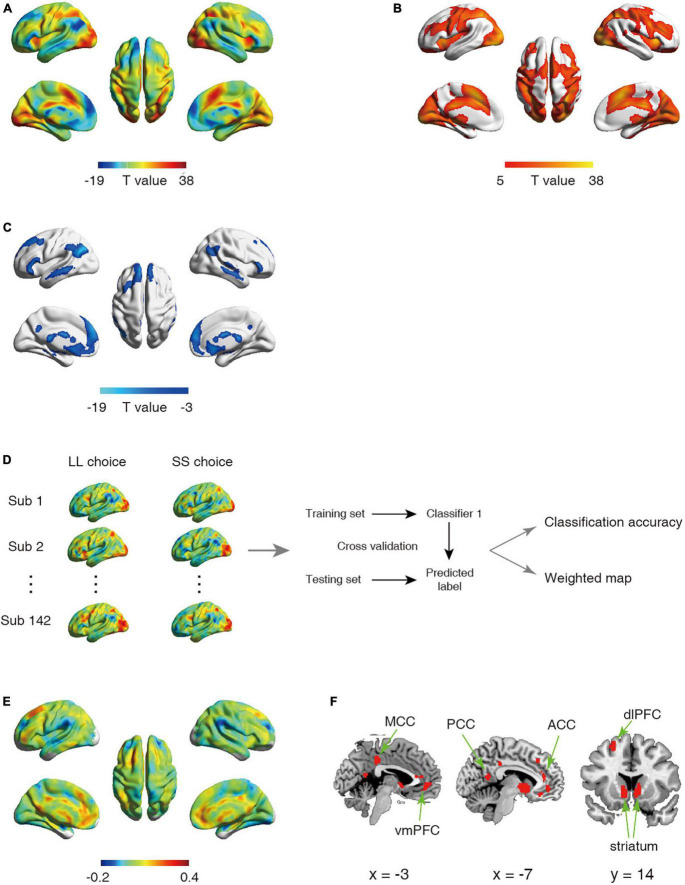
**(A–C)** Statistical activation t-map in univariate analysis. **(A)** Statistical activation t-map for contrast LL vs. SS in univariate analysis, with warm color indicating stronger activation for LL relative to SS (regions exhibiting LL > SS were shown in **(B)** and cool color indicating stronger activation for SS relative to LL (regions exhibiting SS < LL were shown in **(C)** (Significant threshold: p_FWE_ < 0.05). **(D–F)** Schematic overview of analysis for first classifier and corresponding results. **(D)** The univariate analysis was first used to obtain the activation maps for larger later choice (LL choice) and small sooner choice (SS choice) from each participant. The whole-brain activated parametric values from the LL choice and SS choice activation maps for each participant (*n* = 142) were extracted as the features, which were then used to build classifier. **(E)** The whole-brain weighted map. The color bar indicates weight value. **(F)** The top 1% voxels with highest weights (absolute value). Specially, all these voxels were with positive weights.

**FIGURE 2:**
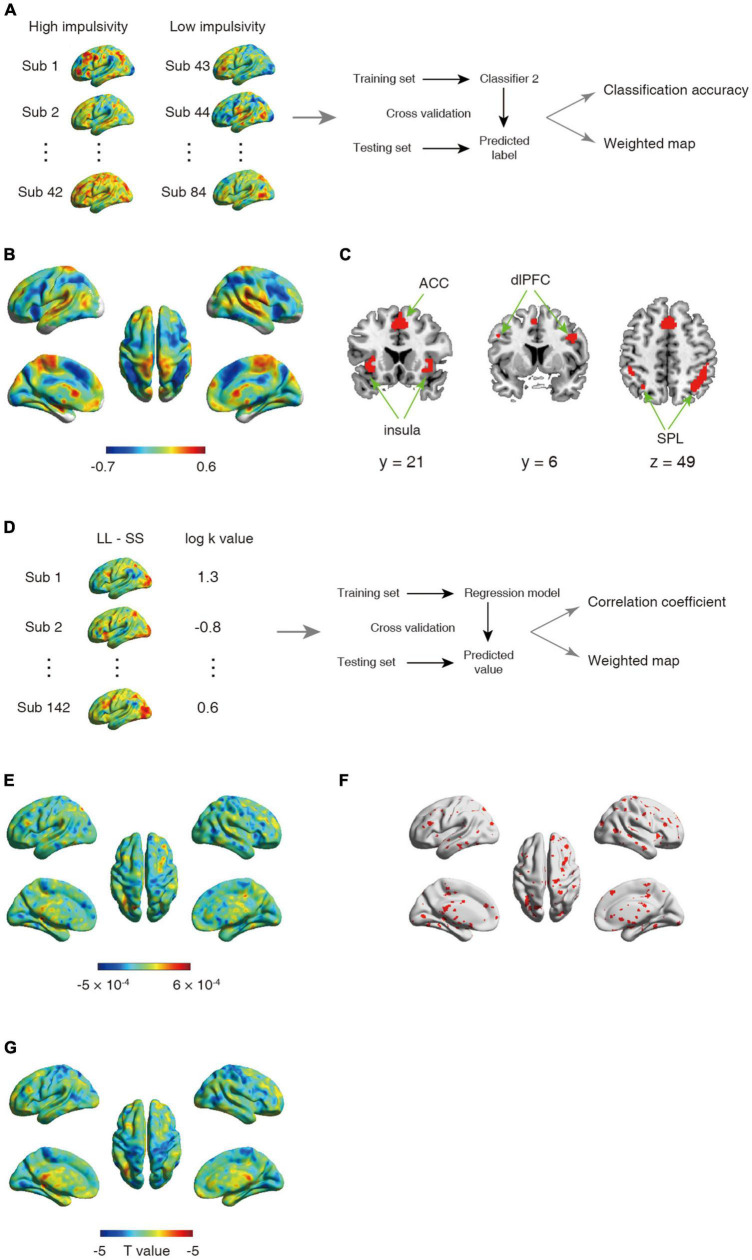
**(A–C)** Schematic overview of analysis for second classifier and corresponding results. **(A)** The univariate analysis was first used to obtain the activation map for contrast between larger later choice and small sooner at the individual level. The whole-brain activated parametric values from the univariate activation map for high (*n* = 42) and low (*n* = 42) impulsive participant were extracted as the features, which were then used to build classifier. **(B)** The whole-brain weighted map. The color bar indicates weight value. **(C)** The top 1% voxel with highest weights (absolute value). Specially, all these voxels were with negative weights. **(D–F)** Schematic overview of a support vector regression (SVR) analysis and the corresponding results. **(D)** The univariate analysis was first used to obtain the activation map for contrast between larger later choice and small sooner at the individual level. The whole-brain activated parametric values from the univariate activation map for all participant (*n* = 142) were extracted as the features, which were then used to build classifier. **(E)** The whole-brain weighted map. The color bar indicates weight value. **(F)** The top 5% voxel with highest weights (absolute value). **(G)** Statistical t-map for correlation between one’s log k-value and brain activations.

A general-linear model (GLM) approach was used to estimate the task events. The GLM had two regressors of interest: (1) trials in which the LL choice was chosen (LL choice); (2) trials in which the SS choice was chosen (SS choice). These two regressors were convolved with the canonical hemodynamic response function. In addition, six raw head-movement parameters were included in model as nuisance regressors. A high-pass filter of 1/128 Hz was implemented to remove low frequency drift from the time-series. We computed first-level contrasts as the following: (1) SS, (2) LL, (3) LL > SS. The contrast maps were then used as inputs in the following second-level univariate analyses and multivariate pattern analyses. For the univariate analyses, effects (see Figures 1B, C, 2G) were corrected for multiple comparisons using family-wise error rate (FWE) correction with a *p*-value set to 0.05.

First, we conducted two classifications which applied the linear support vector machines (SVM, https://www.csie.ntu.edu.tw/cjlin/libsvm/; model setting: “-s 0 -t 0”) with default parameter [c = 1 (Chang and Lin, 2011)]. To facilitate classification across participants, each feature (i.e., parametric value of each voxel) was normalized across the training set, and the normalization parameters were applied for normalizing the test set. Specifically, the first classifier was employed to assess the difference in the neural representation between SS choice and LL choice, which draw upon the contrast images of SS (contrast 1) and LL (contrast 2) extracted respectively for each participant, resulting in two images per participant. We conducted a leave one-subject-out cross validation procedure across participants (i.e., exclude the 2 images from one participant for testing, train with the remaining 282 images from 141 participants) to estimate the predictive capability of neural encoding patterns for intertemporal decision-making (Figure 1D).

The second classifier was used to evaluate the difference of neural representation between high impulsive participants and low impulsive participants in the contrast LL > SS (contrast 3), resulting in one image per participant. Differed from the first classification, the cross validation procedure was performed by excluding two contrast images from two participants for testing and training with the remaining 82 contrast images from 82 participants, with 41 being high impulsive and 41 being low impulsive, to estimate the predictive capability of neural encoding patterns for intertemporal decision-making of high and low impulsive participants (Figure 2A). Note that for both the first and the second classifications, we also applied 10-fold cross-validation strategies to verify the stability of the results.

In addition, to control for the influence of age difference between the high and low impulsive participants, we further regrouped the 84 participants in the second classification based on their age rather than their discount rate. Otherwise, procedure identical to the second classification was employed to estimate the predictive capability of neural encoding patterns for older and younger participants’ intertemporal decision-making.

In these classifications, the classifier was trained in the training set and then applied to the test set to obtain the labels of untested images. After all rounds of cross validation were completed, averaged classification accuracy was calculated to quantify the classifier performance. To determine whether the accuracy was significantly higher than values expected by chance, we performed a permutation test that the task labels were randomly shuffled 1,000 times and ran the above prediction pipeline each time. Based on a null distribution of the accuracy, we estimated the significance by dividing the number of permutations that showed a higher value than the actual accuracy by the total number of permutations.

Specially, to examine the predictive capability of neural encoding patterns for discount rates of all participants, we performed another analysis using support vector regression (SVR) based on data from 142 participants. By using a leave one-subject-out cross validation, one participants was used as the testing sample, and the remaining participants were used as the training samples to select the features and build the model. This processing was repeated until all participants became a testing sample. After all folds were completed, we obtained the predicted log k value for each participant. Pearson’s correlation coefficient (*r*) was calculated based on the linear interdependence between the predicted value and the actual value. To assess the significance, we randomly shuffled the log k values 1,000 times and ran the above prediction procedure for each time to obtain a null distribution of correlation coefficients between the predicted and actual log k values. Significance was caculated by dividing the number of permutations that showed a higher value than the actual coefficient by the total number of permutations.

In order to identify the brain regions made the most prominent contributions in the classifier, we trained a model using images from all analyzed participants to obtain the weight component for each voxel (Haufe et al., 2014; Cui et al., 2020; He et al., 2021). A higher absolute value of the weight indicates a greater contribution of the corresponding feature to the classification.

## Results

### Behavioral results

The average delay discount rates (log k) were −1.78 ± 0.33 (mean ± SD). The log k was −1.43 ± 0.11 in high impulsive group (*n* = 42) and −2.21 ± 0.19 in low impulsive group (*n* = 42), respectively.

### Neural pattern differentiates different intertemporal choices

Univariate analysis revealed that in line with previous studies (McClure et al., 2004, 2007), SS choice led to stronger activations in PCC, vmPFC, striatum relative to LL choice (Figures 1A–C and Tables 1, 2). On the other hand, we employed the first classifier to estimate the predictivity of neural encoding patterns for intertemporal decision-making. The results indicated that the classifier was able to efficiently differentiate individuals’ intertemporal decisions between immediate reward options and the delayed ones with classification accuracy as high as 76.06% (*p* < 0.001, 1,000 permutation tests). The top 1% high weighted voxels located in the value network [vmPFC (MNI coordinate of peak voxel: 8, 38, −8), bilateral ventral striatum (−10, 6, −4; 10, 6, −4), PCC (−4, −54, 14)] as well as cognitive control network [dlPFC (−22, 28, 46), anterior cingulate cortex (ACC, −6, 38, 12), mid-cingulate cortex (MCC, −4, −34, 38)], all with positive weights (Figures 1E, F), indicating that these regions play crucial roles in the representation of one’s intertemporal choices. This classification cannot be fully attributed to activation differences, as comparisons between the activation map and weight map showed that vmPFC exhibits stronger activation for SS choice, while bilateral ventral striatum, ACC and MCC exhibiting stronger activations for LL choice, and dlPFC did not show preferential activation for LL or SS. Moreover, we applied a 10-fold cross-validation strategy to verify the stability, which yielded similar results [accuracy: 75.35%, the top 1% high weighted voxels located in the value network [vmPFC (MNI coordinate of peak voxel: −2, 46, −6), bilateral ventral striatum (−6, 8, −6; 8, 8, −4), PCC (−4, −54, 14)) and cognitive control network (dlPFC (−22, 24, 48), anterior cingulate cortex (ACC, −8, 36, 16), mid-cingulate cortex (MCC, −2, −34, 38)], Supplementary Figures 1A, B].

**TABLE 1:** Brain regions showing significant larger activation in the contrast of LL > SS.

Region	Cluster size	MNI coordinates (mm)			*t*-value
		**x**	**y**	**z**	
Occipital lobe	92,632	36	−76	−10	37.06
		18	−90	6	36.17
		−16	−86	−10	36.05
Orbitofrontal cortex	1,191	24	38	−20	13.68
		20	70	0	8.62
		30	62	−12	7.94
Orbitofrontal cortex	662	−22	42	−18	13.03
		−18	52	−20	8.43
		−34	60	−14	7.16
Superior temporal gyrus	62	44	−26	−4	8.24
Paracentral lobule	50	−16	−34	50	7.26
Superior temporal gyrus	71	48	−40	14	7.15
		60	−38	18	5.75
Fusiform	28	−28	−6	−36	7.12

**TABLE 2:** Brain regions showing significant larger activation in the contrast of SS > LL.

Region	Cluster size	MNI coordinates (mm)			*t*-value
		**x**	**y**	**z**	
Medial frontal gyrus	13,059	20	−42	16	18.48
Parietal lobe	2,013	−50	−66	30	15.94
Middle temporal gyrus	1,382	62	−14	−12	10.90
Superior temporal gyrus	1,012	60	−58	28	11.90
Inferior frontal gyrus	457	52	36	−2	11.58
Parahippocampa gyrus	254	22	−12	−16	9.95
Insula	241	44	−12	−4	10.44
Insula	89	−42	−4	−12	6.85
Parietal lobe	62	−8	−52	30	7.10

### Neural pattern in cognitive control network distinguishes participants with high and low impulsivity

Substantial individual differences of one’s discount rate have been widely reported (Figner et al., 2010; Peters and Buchel, 2011). To test whether and where the ensemble activities in our brain reflected this difference, we performed another classification to discriminate the same contrast images (LL > SS) from impulsive and patient individuals. The results showed that albeit the decision contrasts were held invariant, contrast images were robustly classified into the relevant groups (accuracy = 91.67%, *p* < 0.001, 1,000 permutations). The top 1% high weighted voxels situated primarily in cognitive control network [bilateral insula (−26, 22, −2; 32, 33, −4), bilateral superior parietal lobe (SPL, −40, −42, 44; 44, −42, 46), bilateral dlPFC (−46, 4, 36; 40, 4, 32), and ACC (2, 20, 42)], all with negative weights (Figures 2B, C). These results were verified by a 10-fold cross-validation [accuracy: 92.86%, the top 1% high weighted voxels situated primarily in cognitive control network [bilateral insula (−34, 18, −4; 34, 22, −4), bilateral superior parietal lobe (SPL, −30, −62, 46; 34, −58, 46), bilateral dlPFC (−46, 6, 36; 46, 6, 32), and ACC (0, 18, 46)], Supplementary Figures 1C, D]. On the other hand, univariate analysis found no brain region’s activation was strongly correlated with one’s discount rates (threshold: p_*FWE*_ < 0.05, Figure 2G).

Another analysis using SVR, which drawed upon data from all 142 participants showed a weak yet significant correlation between the predicted log k value and the actual log k value (*r* = 0.16, *p* < 0.01, 1,000 permutations), indicated that one’s discount rate can be predicted from one’s neural activity. Specifically, all abovementioned regions (resulted from the second classification using SVM) were included in the top 5% high weighted voxels obtained from the SVR analysis [bilateral insula (−36, 17, 0; 35, 17, 0), bilateral superior parietal lobe (−34, −60, 42; 41, −43, 49), bilateral dlPFC (−44, 0, 38; 48, 10, 40), and ACC (1, 12, 52), Figures 2D–F].

According to previous evidence from adolescent, discount rate exhibited a modest but significant decrease to age (Anokhin et al., 2015). In this sample, participants with high and low impulsivity differed in age [i(82) = 2.24, *p* = 0.028]. To determine whether the successful classification above was based on disparity in age, we regrouped the sample (*n* = 42 in each group) based on their age [*t*(82) = 13.55, *p* < 0.001] and trained a classifier to discriminate the same contrast images (LL > SS) from older and younger individuals. The results showed that the classifier was unable to successfully classify the contrast image into the corresponding age group (accuracy = 45.24%, *p* = 0.72, 1,000 permutation tests), suggesting that the successful classification of patient and impulsive individuals was unlikely due to the age difference between the two groups.

## Discussion

Using multivariate pattern analysis, the present study found that the spatially distributed information of neural activity in human brain can not only robustly predict one’s intertemporal choice, but also successfully differentiate high and low impulsive participants with high accuracies while controlling for age. Notably, in addition to regions from value network that have been reported to preferentially respond to immediate rewards, our results showed that the neural representation of different intertemporal choices from regions that were previously suggested to have comparable ensemble activities (McClure et al., 2004), were actually dissociable. These findings provide clear evidence that substantiate the important role of value and control networks in the neural representation of individual intertemporal decision.

Although the neural mechanisms between different choices processes in intertemporal decision-making have been explored extensively (Kable and Glimcher, 2007; Hare et al., 2009; Harris et al., 2011; Waegeman et al., 2014; Maier Silvia et al., 2015), relatively less studies have examined such different neural mechanisms from the perspective of neural representation (Wang et al., 2014, 2021; Chen et al., 2019; Piva et al., 2019). By taking full advantage of information from the voxels, multivariate analysis endows us with higher sensitivity and reliability (Lv et al., 2021). Our study benefits from such advantages, as besides confirming dissociable pattens in regions previously reported to have different activation levels, we identified a range of regions with distinct distributed ensemble activities that have been documented as overall equally activated.

In line with the previous finding (Chen et al., 2019), the classifier showed that global neural representation could distinguish the LL and SS choice with high classification accuracy at the whole brain level. Specially, by quantifying feature weight for each voxel, we further demonstrated that several regions play critical roles in the classification. As aforementioned, prior research on neural substrates of intertemporal choices with the help of univariate analysis emphasize the participation of value network (McClure et al., 2004, 2007), which are replicated in our results. Specifically, the value network system, including medial prefrontal cortex, striatum, PCC, which are considered as one of the potential neural substrates of choice impulsivity, have been reported to respond stronger to immediate rewards (McClure et al., 2004, 2007; Hare et al., 2008, 2011). Furthermore, they track the subjective valuation of delayed rewards, as activities in these regions increase along with the increase of objective amount of a reward and decrease of the imposed delay to a reward (Kable and Glimcher, 2007). The representation of relative subjective value in the dorsomedial prefrontal cortex (dmPFC) is further confirmed with multivariate pattern analyses (Wang et al., 2014; Piva et al., 2019).

Critically, by identifying the prominent roles regions such as ACC, MCC, dlPFC play in the representation of ultimate choice, our results additionally highlight the role of cognitive control network in the representation of ultimate choice. As nodes of cognitive control network (Monosov et al., 2020; Domic-Siede et al., 2021), albeit these regions were seldom reported to vary in mean fMRI responses of different choices (McClure et al., 2004, 2007), they have been reported to be preferentially activated in difficult choice trials (McClure et al., 2004; Jimura et al., 2018) and shorter delay time (Wang et al., 2021). Additionally, recruitment of cognitive control system has also been reported in other value-based decision making tasks (Lee and Daunizeau, 2021; Matsui et al., 2022). Together with a previous study (Chen et al., 2019), our results corroborated these findings and took a step further with the help of multivariate analysis to demonstrate an integrative framework of intertemporal choice, in which value system as well as control system are implemented (Berns et al., 2007).

Considerable individual differences have been repeatedly described in delay discounting, which reflect crucial facets of behavioral impulsivity and self-control (Figner et al., 2010; Peters and Buchel, 2011). In parallel, imaging studies investigating neural correlates of intertemporal choice in impulsive and patient individuals showed that they exhibited various neural dynamics even under identical choice condition (Jimura et al., 2013, 2018). Specifically, the relationship between the recruitment of dlPFC and choice difficulty was primarily observed in low impulsive individuals. Activities in ventral striatum and anterior prefrontal cortex correlated negatively and positively, respectively, with the degree of discounting. Most of the existing works using multivariate approach to investigate the individual differences in decision impulsivity draw upon brain activity during resting state such as regional homogeneity pattern and functional connectivity (Lv et al., 2019; Wang et al., 2020). Using functional imaging data from a relatively large sample, our results indicate that although decision contrast remained the same (LL > SS), drastic distinct patterns were found in individuals with different discount rates, indexed by high accuracy when decoding high and low impulsive participants. This was verified by a SVR analysis based on data from 142 participants. Furthermore, regions in cognitive control network take leading roles in differentiating the representations of impulsive and patient participants. Despite being modulated by reward type and state of deprivation, discount rate is relatively stable over time (Kirby, 2009), hence can be regarded as an attribute of the person. Our results mapped this characteristic of individual onto one’s neural activities.

Nevertheless, we don’t exclude the involvement of value system in one’s discount rate, as we only reported the top 1% high weighted voxels of classification, leaving clusters from regions such as ventral striatum and PCC unexamined. In addition to being important nodes of cognitive control network, aforementioned regions such as ACC and anterior insula also engage in the encoding of value (Norbury et al., 2018; Yee et al., 2021). Indeed, ACC and anterior insula also play an important role in distinguishing participants with different impulsivities, suggesting that value system and control system are likely to act in concert in one’s intertemporal choice, substantiating that patient decision requires the individual is both willing and able to wait (Roberts and Fishbach, 2022). On the other hand, delay-discounting rate was successfully predicted from representational connectivity between regions representing the amount of reward (dmPFC, vmPFC, lateral frontal pole cortex, etc.) (Wang et al., 2021), suggesting that connections in the value system also play an important role in one’s discount rate.

It’s worth noting that a growing body of research link maladaptive intertemporal choices and psychiatric illnesses such as substance abuse, gambling disorder, making it being proposed as a candidate behavioral marker for psychopathology (Bickel, 2015). In parallel, alternations were documented in brain structure and functional connectivity associated with value and cognitive control system in individuals with impulsive problems (Weng et al., 2013; Freinhofer et al., 2020). With the help of multivariate analysis, our study also identified functional neural signatures in cognitive control network corresponding to one’s impulsivity, which underscores cognitive control as a potential mechanism underlying various impulsive disorder, making these regions promising therapeutic target for altering impulsive behaviors. Previous study took advantage of repetitive transcranial magnetic stimulation and found that disruption of function of the lateral prefrontal cortex increased choices of immediate rewards over larger delayed rewards (Figner et al., 2010). Moreover, efforts have been made to enhance cognitive performance to shift behavior away from immediate and risky rewards in non-clinical individuals, albeit with little success (Kable et al., 2017), which may be due to the training population and targeted cognitive domain. Cognitive control training in clinical population is warranted to explore the candidate therapy in ameliorating impulsivity.

Some issues warrant further discussion. First, this study includes only young and healthy adults. Previous research found that discount rate is related to one’s age, exhibiting a decrease to older adults (Anokhin et al., 2015). Future research will need to establishe the link between changes in neural activity patterns and changes in age-related impulsivity. Secondly, numerous research links abnormal discount rate with psychiatric disorder such as substance abuse and gambling disorder (Bickel et al., 1999; Alessi and Petry, 2003). It would be interesting to include participants with these psychiatric disorders in the furture.

In summary, this study adopted multivariate analysis approach in a relatively large sample to explore the underlying neural substrates of impulsive choice. At the global level, the classifier built with global activation pattern not only successfully distinguished the intertemporal choices, but also effectively distinguished the high and low impulsive participants. Furthermore, the results underlined regions in value network and cognitive control network as critical components of these neural signatures. These results deepen our understanding of the neural correlates of intertemporal choice as well as emphasize the utility of pattern analysis in predicting intertemporal decision-making and individual differences.

## Data availability statement

The original contributions presented in this study are included in the article/Supplementary material, further inquiries can be directed to the corresponding authors.

## Ethics statement

The studies involving human participants were reviewed and approved by the University of Pennsylvania Institutional Review Board. The patients/participants provided their written informed consent to participate in this study.

## Author contributions

Both authors undertook the data analysis and wrote the manuscript.

## Footnotes

1
https://www.fil.ion.ucl.ac.uk/spm12/

## Supplementary material

Supplementary Figure 1 Corresponding results of classifications using 10-fold cross-validation strategies. **(A)** The whole-brain weighted map of the first classifier (classify LL vs. SS) and **(B)** the top 1% voxels with highest weights (absolute value). Specially, all these voxels were with positive weights. **(C)** The whole-brain weighted map of the second classifier (classify high impulsive individual vs. low impulsive individual) and **(D)** the top 1% voxels with highest weights (absolute value). Specially, all these voxels were with negative weights. The color bar indicates weight value.
