# Supplementary figures and images for: Multivariate analysis differentiates intertemporal choices in both value and cognitive control network
Source: Front Neurosci. 2023 Feb 28;17:1037294. doi: 10.3389/fnins.2023.1037294 (PMC10011120; doi:10.3389/fnins.2023.1037294)

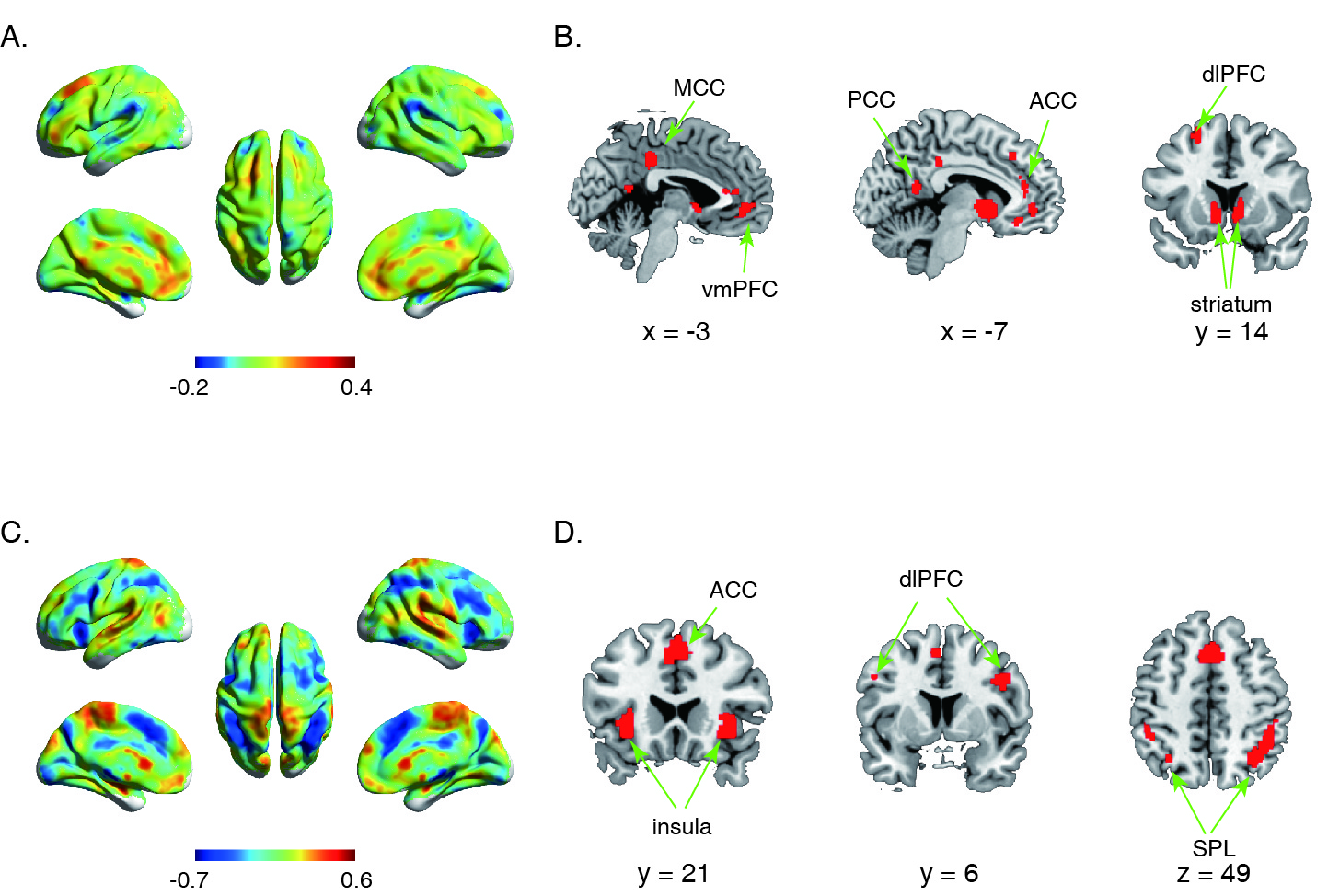

Supplement: Supplementary Figure 1 — Corresponding results of classifications using 10-fold cross-validation strategies. (A) The whole-brain weighted map of the first classifier (classify LL vs. SS) and (B) the top 1% voxels with highest weights (absolute value). Specially, all these voxels were with positive weights. (C) The whole-brain weighted map of the second classifier (classify high impulsive individual vs. low impulsive individual) and (D) the top 1% voxels with highest weights (absolute value). Specially, all these voxels were with negative weights. The color bar indicates weight value. [file Image_1.JPEG]
